# Knowledge, Attitudes, and Practices about the Prevention of Mosquito Bites and Zika Virus Disease in Pregnant Women in Greece

**DOI:** 10.3390/ijerph14040367

**Published:** 2017-03-31

**Authors:** Varvara A. Mouchtouri, Dimitrios Papagiannis, Antonios Katsioulis, Georgios Rachiotis, Konstantinos Dafopoulos, Christos Hadjichristodoulou

**Affiliations:** 1 Department of Hygiene and Epidemiology, University of Thessaly, Larissa 41222, Greece; mouchtourib@med.uth.gr (V.A.M.); dpapajon@yahoo.gr (D.P.); akatsioul@med.uth.gr (A.K.); grachiotis@gmail.com (G.R.); 2 Department of Obstetrics and Gynecology, University of Thessaly, Larissa 41222, Greece; kdafop@yahoo.com

**Keywords:** vector-borne infections, viral infections, pregnancy, Zika virus, Knowledge, Attitudes, and Practice (KAP) study, pregnant, repellent

## Abstract

A survey among 573 pregnant women in Greece was conducted through self-completion of a questionnaire in July 2016. Traveling abroad the last six months was declared by 10.5% and 13.0% of pregnant women and their male sex partners, respectively, while 77.4% (441/570) had heard about Zika virus disease (ZVD). A lack of knowledge about sexual transmission of ZVD was identified in 63.3% of pregnant women, and 24.1% of responders did not know the risks to the fetus and baby. Approximately 73% of responders believed that the mosquito bites can affect their fetus and baby and 18% did not take measures to prevent mosquito bites routinely. Multivariable logistic regression models showed that traveling abroad the last six months by pregnant women correlated with correctly answering the question about the transmission of ZVD through bites of infected mosquitoes (Odds Ratio, OR = 10.47, 95% CI = 1.11–98.41). Traveling abroad with a male sex partner over the last six months correlated (OR = 2.05, 95% CI = 0.99–4.23) with responding correctly to the four key questions about the transmission of ZVD through mosquito bites, the risk of microcephaly, and the risks of traveling to the affected countries. A score of ≥5 for the nine responses given to questions of knowledge and attitudes was associated with a Bachelor of Science degree (OR = 1.54, 95% CI = 1.09–2.18), antenatal care at a public hospital (OR = 2.26, 95% CI = 1.28–3.98), being a civil servant as occupation (OR = 1.96, 95% CI = 1.10–3.48), and having gotten information about ZVD from the public health sector (OR = 2.04, 95% CI = 1.05–3.98). In conclusion, we found considerable knowledge gaps related to ZVD among Greek pregnant women. These study results are useful in targeting pregnant women for the prevention of potential Zika virus infections.

## 1. Introduction

Zika virus disease (ZVD) is a mosquito-borne and sexually transmitted disease [1]. Infected mosquitoes of the *Aedes* species can transmit the virus to humans through bites. There is evidence for an association of infection during pregnancy with congenital central nervous system malformations of the developing fetus, as well as Guillain–Barré syndrome in adults, and rarely in children. Pregnant women are considered most at risk for disease prevention strategies [2].

In Europe, according to the European Center for Disease Prevention and Control (ECDC), travel-associated ZVD has been reported by 19 countries, and reports include cases among pregnant women, but no autochthonous mosquito-borne transmission has been reported in European Union countries in continental Europe [3]. ECDC in its 19th risk assessment estimated that “given the low vector competence of the studied European populations of *Aedes albopictus*, the likelihood of local vector-borne transmission in the EU is considered to be low to moderate during the summer period” [3,4].

Greece is an unaffected, but receptive area for Zika virus transmission, since *Aedes albopictus* mosquitoes have been established in continental areas and some islands [5,6]. Among other measures implemented in Greece, information about preventative measures of the Zika virus disease (ZVD) is given to the traveling population to or from the affected countries with emphasis on women who are pregnant or of childbearing age or want to conceive, as well as their partners [7].

The Emergency Committee under the International Health Regulations in its fifth meeting regarding microcephaly, other neurological disorders, and the Zika virus declared that the temporary recommendations including travel advice should be part of the long-term response mechanisms and declared the end of the Public Health Emergency of International Concern [8]. Part of the long-term preparedness and response planning of countries are measures to prevent ZVD in pregnant women [2,6].

ZVD received high publicity in the mass media, especially before and during the 2016 Olympic Games [9,10]. Official travel advice provided by the Greek Center for Disease Control and Prevention is available on their website [7]. This study attempted to examine the knowledge, attitudes, and practices (KAPs) about mosquito bite prevention and about ZVD in pregnant women in Greece in July 2016.

## 2. Materials and Methods

### 2.1. Survey Questionnaire

A questionnaire was composed considering the resource pack of the World Health Organization (WHO) for designing and implementing KAP surveys for ZVD and potential complications [11]. The questionnaire was designed by a team of experts consisting of an epidemiologist, an occupational health professional, a pharmaceutical representative, a gynecologist, and a public health specialist. A pilot test of the first draft of the questionnaire took place in one gynecologist's office, where distribution, self-completion by 10 pregnant women, and then data collection and analysis took place. The results of the pilot testing were considered in order to further modify the distribution protocol and the questionnaire. A second pilot test followed and then the questionnaire and the database were finalized. The survey questionnaire consisted of 24 questions about (a) demographic characteristics and information for recent travel; (b) knowledge (ZVD cause and prevention); (c) attitudes; and (d) practices (mosquito bites prevention and sexual and reproductive health). The list of questions included can be found in the Supplementary Materials.

The questionnaires were distributed to gynecologists’ offices together with a cover letter explaining the data collection protocol including the survey background, objectives, methods, data use, confidentiality, and anonymity issues, as well as the fact that participation in the survey was voluntary. The doctor and his or her staff were given instructions to ensure independent completion of the questionnaire and privacy at the time of completion. The hard copy of the questionnaire, after verbal consent to participation in the survey was attained, was given to the pregnant women for self-completion in the waiting room. After anonymous completion, the questionnaire was enclosed in an envelope. The gynecologist was available to provide information about disease risks, prevention, and control and to answer any questions about ZVD the responder or her partner might have after completion of the questionnaire. The study was approved by the Steering Committee of the Postgraduate Program of Applied Public Health and Environmental Hygiene of the Medical Faculty, University of Thessaly (Second Assembly 2016; Project Identification Code 01072016).

### 2.2. Sampling

The target population included pregnant women living in Greece, while the sampling population was pregnant women attending a gynecologist in July 2016.

Geographically stratified cluster sampling was implemented considering country administrative regions (Eurostat’s NUTS-2) as strata and gynecologists as clusters (primary sampling unit). Within each region, the doctors were selected by simple random sampling, and their number was proportional to strata size (distributed proportionally to women population aged 15–49 in the 2011 census within each strata).

A sample size around 535 pregnant women was required to detect a finding of 50% with a precision of ±6% for 95% Confident Intervals (CIs), a power of 80% at a two-sided 5% level of statistical significance, and a design effect of 2. Accounting for the initial sample size and an 80% and 90% response rate of gynecologists and pregnant women, respectively, the final sample size required was calculated to be about 745 pregnant women. Fifty-six gynecologists were randomly selected and asked to disseminate the questionnaire to their patients from 1 July to 31 August 2016. It was estimated that 20 questionnaires would be collected from each gynecologist in the Attica region, 15 in Central Macedonia, and 10 in the rest of the administrative regions.

### 2.3. Data Management

A database was developed using the lime survey software [12] at the University secured server. Data were entered in the database by two persons after receiving training for the data entry process. Each participant was assigned a code number for data entry and analysis. Anonymity and confidentiality was ensured through the data collection, entry, analysis, and storage. To ensure de-identification of the survey, the questionnaire did not include any name, address, zip code, date of birth, nor doctor’s name or town. The demographic characteristics included age, region of residence, and nationality. It was not possible to re-identify the surveys from the completed hard copies or from the database created for data analysis purposes.

### 2.4. Scoring of Responses

Each correct response to a knowledge question was scored with one point. The first aggregate score representing ZVD knowledge was created by adding one point for every correct response to Questions 1–7 and Questions 16 and 17 (Supplementary Materials). A second aggregate score representing key ZVD knowledge was created by adding one point for every correct response to Questions 1–3 and Question 7 (Supplementary Materials).

### 2.5. Statistical Analysis

Qualitative variables are presented as frequencies with either percentages or 95% CI, or both.

For a univariate analysis, the chi-square test was applied to associate demographic characteristics and other factors with KAP responses as well as scores of responses to knowledge questions calculating the relative risks (RR) with corresponding 95% CI.

Multivariable logistic regression models were used to identify independent risk factors for the KAPs and scores of responses to knowledge questions to calculate the odds ratios (OR) and the corresponding 95% CI. Factors with a *p*-value less than 0.200 in univariate analysis were included in multivariable analysis.

A result with a *p*-value < 0.05 was considered to be statistically significant. All statistical analyses were conducted taking into the account the clusters of the study through the complex sample module of SPSS 19.0 (IBM SPSS Inc., Armonk, NY, USA).

## 3. Results

Of the 56 gynecologists selected for the survey, 44 participated in the study (doctor’s response rate 78.4%) to whom 605 questionnaires were sent. In total, 573 questionnaires were completed by pregnant women (response rate 94.7%). The total survey response rate was 74.2%.

Of the 573 responders, 540 were Greek (94.2%) and others were Albanian, Bulgarian, Georgian, Dutch, Russian, and Czech. Twenty-five were of unknown ethnicity. Demographic characteristics of the study participants can be found in Table 1. The mean and median gestational weeks were 22.8 and 23, respectively (standard deviation: 9.5; min.: 4, max.: 40; 25th, 50th and 75th percentiles: 15, 23, and 31 gestational weeks). 

The majority of pregnant women (511/571, 89.5%) or their male sex partners (496/570, 87%) had not traveled abroad, and of those who traveled abroad (Table 1), only one and her male sex partner visited an affected country that that was categorized by the ECDC as a country with sporadic transmission (no more than 10 locally transmitted cases have been reported in a single area within the last three months) [13].

Of the total 570 pregnant women, 441 (77.4%) had heard about ZVD. Of those 441 responders, 332 were informed from television or radio (75.3%), 38 from a physician (8.6%), 157 from the Internet (35.6%), and 13 from a public health institution webpage (2.9%). The majority of pregnant women judged the information they have about ZVD as inadequate (464/573, 81%), while only 75 (13.1%) rated the information as adequate.

Table 2 presents the responses to knowledge and attitudes questions. Of the total 544 responders, 186 (34.2%) said that, if their male sex partner returned from a trip to a country where many cases of ZVD have occurred, they would have sexual intercourse without any protection. Of the 358 pregnant women who responded that they would have sexual relations with protection, 46 (16%), 31 (10.8%), 59 (20.6%), and 87 (30.3%) responded that they would take protection for 2, 3, 4, or 6 weeks after traveling respectively.

A total of 54 responders answered correctly to all four key questions (9.4%), while 55 responders (9.6%) did not provide any correct answer.

A total of 231 responders received a score of ≥5 (40.3%) on questions regarding knowledge and attitudes, while 31 responders (5.4%) did not answer any of the nine questions correctly.

The correct answers to individual KAP questions were associated with the following factors by using univariate analysis and multivariable logistic regression models: demographic characteristics (age ≥30, high or medium income), being a pregnant woman—or her male sex partner—that has traveled abroad in the past six months, to which sector one’s pregnancy care provider (public hospital) belonged, and the source of information about ZVD (public health sector).

The results of univariate analysis are shown in Table 1.

Multivariable logistic regression models were used to test for an association between individual question responses with the above-mentioned factors. This analysis showed that, for pregnant women, traveling abroad (to any country) over the last six months correlated with correctly answering the question about the transmission of ZVD through bites of infected mosquitoes (OR = 10.47, 95% CI = 1.11–98.41), but no correlation was found between traveling abroad and correctly answering the question about the sexual transmission of ZVD (OR = 1.17, 95% CI = 0.79–1.73, *p*-value = 0.445). The first aggregate score of ≥5 and the second aggregate score of 4 were associated with the factors with a *p*-value < 0.2 in the univariate analysis. A score of ≥5 in the first aggregate score questions correlated with having a Bachelor of Science degree (OR = 1.54, 95% CI = 1.09–2.18), having antenatal care at a public hospital (OR = 2.26, 95% CI = 1.28–3.98), having received information about ZVD from the public health sector (OR = 2.04, 95% CI = 1.05–3.98), and being a civil servant as an occupation (OR = 1.96, 95% CI = 1.10–3.48). Providing correct answers to the four key questions (second aggregate score questions) correlated with women aged ≥30 years old (OR = 2.83, 95% CI = 1.13–7.07) and their male sex partner’s having traveled abroad in the last six months (OR = 2.05, 95% CI = 0.99–4.23).

## 4. Discussion

This study showed that most of the pregnant women who participated in the survey were not well informed about all transmission modes and risks of ZVD. Interestingly, the majority of participants (75%) were informed about ZVD from television or radio but lacked knowledge about the sexual transmission of the virus (63.3%), while more than half of them (53%) were not aware of the risks of congenital malformations. Despite the publicity that this public health emergency of international concern received, especially before and during the summer of 2016 due to the Olympics in Brazil, more than half of the responders lacked important knowledge about ZVD. Information campaigns through the mass media can reach a wide audience but must ensure that those media are trusted and are giving complete and balanced messages to the public. More importantly, pregnancy care providers play a key role in such prevention strategies [14,15]. A study examining knowledge of ZVD among 112 pregnant women who were in areas with active transmission showed that all of them knew about the mosquito-borne transmission of ZVD, but 93% knew about the sexual transmission and 89.3% were aware of the link of ZVD during pregnancy and birth defects [16].

Pregnant women in Greece who traveled abroad (not only to the affected countries) were better informed about the risks of ZVD transmission through mosquito bites compared to pregnant women who did not travel abroad (OR = 10.47, *p*-value = 0.04). This fact can be attributed to the effectiveness of information campaigns implemented in the summer of 2016 targeting the traveling population. Correct knowledge of the four most important key issues for the transmission and prevention of the disease demonstrated by pregnant women who were of the age of ≥30 years and those whose male sex partner had traveled abroad. Factors including having a Bachelor’s degree, having searched for information from accurate sources from public health authorities or the doctor, having a pregnancy care provider from a public hospital, and being a civil servant associated with a higher score of correct knowledge in all questions. This could be explained by the fact that pregnant women whose male sex partner traveled recently had received recent, accurate, and complete information, while others answered correctly because they looked for information from accurate resources, while their educational level might play a role as well. Another study conducted among dental practitioners demonstrated correct knowledge in a small percentage of participants (about 38%) and the factor that significantly associated with knowledge was the postgraduate degree [17].

Studies conducted in Puerto Rico, Brazil, and El Salvador showed that ZVD has a higher incidence among women than among men, and the highest rates are reported among women aged 20–49 years, which corresponds to women of childbearing age [18,19,20].

The study population was not targeted to travelers to ZVD-affected countries, so conclusions cannot be made about the effectiveness of information strategies in Greece targeting the traveling population to the affected countries. One participant had traveled to an affected country with her male sex partner and lacked knowledge of the sexual transmission of ZVD, but was aware of the mosquito bite transmission. It would be interesting to evaluate the effectiveness of preventative strategies by KAP surveys among travelers to the affected countries. To our knowledge, three KAP studies have been published for ZVD, and one of them targeted pregnant women in an affected country [16,17,21]. The pack of the WHO for designing and implementing KAP surveys for ZVD and potential complications provides a useful resource for designing and conducting such surveys [11].

Currently, routine health advice to pregnant women does not necessarily include prevention of mosquito bites in non-Zika affected or malaria-free countries, since there is no evidence to justify such strategies. Our study demonstrated that 18% of pregnant women do not take measures for mosquito bite prevention routinely. There are some studies examining the effects of vector-borne diseases other than ZVD and malaria, such as West Nile Virus and Chikungunya, on pregnancy and the fetus [22,23], but further studies might be needed to collect evidence of the potential risks of vector-borne diseases to unborn children. In any case, to protect unborn children, preparedness plans for vector-borne disease prevention among pregnant women should be activated in case local transmission of ZVD is confirmed [6,24,25].

The results of this study are subject to the following limitations: About 40% of the total 92,148 births registered in Greece in 2014 were given in public hospitals [26]. However, due to financial reasons, pregnant women may receive antenatal care from the private sector, albeit giving birth in a public hospital. It was not possible to identify statistics from an official source about the proportion of pregnant women who are provided antenatal care in a public hospital and in the private sector, so we were unable to assess whether the study participants receiving pregnancy care at a public hospital were underrepresented. Further, despite the overall satisfactory response rate (73%), we were not able to obtain data about ZVD knowledge from non-responders, so some selection bias may have occurred.

## 5. Conclusions

Greece is not a ZVD-affected country, so information campaigns mainly target travelers to the affected countries who receive advice about the risks of the disease and its prevention [7]. Greece is a receptive area for Zika virus transmission [6], so preparedness plans to prevent any potential future local transmission should be in place. These study results might be useful for preparedness planning for vector-borne disease, as well as, in particular, for the prevention of potential local transmission of the Zika virus and for designing travel advising strategies to the traveling population to Zika-affected countries.

## Figures and Tables

**Table 1:** Demographic characteristics and information about pregnant women and their male sex partners that have traveled abroad.

Characteristic	Details		Frequency (%)
Age	≥30		408 (71.7)
	<30		161 (28.3)
	Total		569 (100.0)
Region	Attica region		117 (20.4)
	Other regions:	Crete, Southern Aegean, Peloponnese, Central Greece, Western Greece, Ionian Islands, Epirus, Thessaly, Western Macedonia, Central Macedonia, Eastern Macedonia and Thrace	456 (79.6)
	Total		573 (100.0)
Provider of pregnancy care	Private clinic/obstetricians		529 (96.2)
	Public hospital		21 (3.8)
	Total		550 (100.0)
Level of education	Master or Doctor of Philosophy degree		91 (16.0)
	Bachelor degree		288 (50.5)
	Up to secondary education		191 (33.5)
	Total		570 (100.0)
Occupation	Civil servant		77 (13.6)
	Private sector employee		290 (51.2)
	Freelancer		71 (12.5)
	Unemployed-housewife		128 (22.6)
	Total		566 (100.0)
Income	Middle/higher		349 (62.4)
	Low		210 (37.6)
	Total		559 (100.0)
Pregnant traveled abroad the last six months	Yes	Zika Virus Disease affected country (1)	60 (10.5)
		Non Zika Virus Disease affected countries (59)	
	No		511 (89.5)
	Total		571 (100.0)
Male sex partner of the pregnant traveled abroad the last six months	Yes	Zika Virus Disease affected country (1)	74 (13.0)
		Non Zika Virus Disease affected countries (73)	
	No		496 (87.0)
	Total		570 (100.0)

**Table 2:** Knowledge, attitudes, and practices (KAPs) for Zika virus disease and sexual contact protection among pregnant women in Greece.

Knowledge/Attitude/Practice	Number of Responses	
	Strongly Agree/Agree/Yes (%)	95% CI
**Knowledge**		
Zika virus disease is transmitted with sexual intercourse.	187/510 (36.7)	29.9–44.0
Zika virus disease is transmitted through the consumption of contaminated food.	134/505 (26.5)	20.3–33.9
Zika virus disease is transmitted through bites of infected mosquitoes.	465/514 (90.5)	84.0–94.5
Zika virus disease causes severe disease, bleeding, and death.	220/489 (45.0)	38.4–51.7
A pregnant woman can get sick from Zika virus disease.	232/573 (40.5)	34.4–46.8
If a pregnant woman has Zika, she is at risk of miscarriage.	220/573 (38.4)	32.7–44.4
If a pregnant woman has Zika, her fetus/baby is at risk of being born with microcephaly.	270/573 (47.1)	42.7–51.6
Is there any available treatment for Zika virus disease?	12/540 (2.2)	1.3–3.8
Is there any vaccine available against Zika?	20/553 (3.6)	2.2–5.8
**Attitudes**		
I believe that mosquito bites to pregnant women can affect the health of a fetus/baby.	401/551 (72.8)	65.8–78.8
If your doctor recommended everyday use of mosquito repellent lotion, would you use it?	447/563 (79.4)	74.6–83.5
I wouldn’t use mosquito repellent because it might be dangerous for the fetus/baby.	74/116 (63.8)	54.4–72.2
I wouldn’t use mosquito repellent because I don’t believe it can protect myself or the fetus/baby.	21/116 (18.1)	12.5–25.4
I believe that traveling to countries with many cases of the Zika virus disease should not be allowed.	214/552 (38.8)	32.8–45.1
If there were a vaccine available against Zika, would you consider receiving it?	117/558 (21.0)	16.2–26.6
I believe that a pregnant woman can go on holidays to a country where many cases of Zika virus disease have occurred without having any risk.	81/563 (14.4)	9.2–21.8
Would you visit a country where many cases of Zika virus disease have occurred?	4/562 (0.7)	0.3–1.9
Do you take any measures to prevent mosquito bites the summer months during your pregnancy?	455/555 (82.0)	75.5–87.0
I take measures for mosquito bite prevention when I realise that there are mosquitoes around.	233/455 (51.2)	43.3–59.1
I take measures for mosquito bite prevention when I visit places where mosquitoes might be present.	186/455 (40.9)	33.4–48.8
I take measures for mosquito bite prevention every day during the daylight hours.	25/455 (5.5)	3.4–8.7
I take measures for mosquito bite prevention every day during the evening and at night.	38/455 (8.4)	5.5–12.5
I wear covering clothes to prevent mosquito bites.	77/455 (16.9)	12.6–22.4
I use repellents to prevent mosquito bites.	244/455 (53.6)	45.8–61.3
I use mosquito coil/repellent liquid vaporizers/tablet to keep mosquitoes away.	210/455 (46.2)	38.4–54.1

CI: Confidence Interval.

## Supplementary Materials

The following are available online at www.mdpi.com/1660-4601/14/4/367/s1, List of questions for knowledge and attitudes.
