# Supplementary material for: Knowledge, Attitudes, and Practices about the Prevention of Mosquito Bites and Zika Virus Disease in Pregnant Women in Greece
Source: Int J Environ Res Public Health. 2017 Mar 31;14(4):367. doi: 10.3390/ijerph14040367 (PMC5409568; doi:10.3390/ijerph14040367)
Supplement: Supplementary file 1 [file ijerph-14-00367-s001.pdf]

List of questions

**Knowledge**	
1	Zika virus disease is transmitted with sexual intercourse
2	Zika virus disease is transmitted through the consumption of contaminated food
3	Zika virus disease is transmitted through bites of infected mosquitoes
4	Zika virus disease causes severe disease, bleeding and death
5	A pregnant woman can get sick from Zika virus disease
6	If a pregnant woman has Zika, she is at risk of miscarriage
7	If a pregnant woman has Zika, her foetus / baby is at risk of being born with Microcephaly
8	Is there any available treatment for Zika virus disease?
9	Is there any vaccine available against Zika?
**Attitudes**	
10	I believe that mosquito bites to pregnant women can affect the health of the foetus/baby
11	If your doctor recommended everyday use of mosquito repellent lotion would you use it?
12	I wouldn't use mosquito repellent because it might be dangerous for the foetus/baby
13	I wouldn't use mosquito repellent because I don't believe it can protect myself and the foetus/baby
14	I believe that travelling to countries with many cases of Zika virus disease should not be allowed
15	If there was a vaccine available against Zika, would you consider having it?
16	I believe that a pregnant woman can go on holidays to a country where

	many cases of Zika virus disease have occurred without having any risk
17	Would you visit a country where many cases of Zika virus disease have occurred?
18	Do you take any measures to prevent mosquito bites the summer months during your pregnancy?
19	I take measures for mosquito bite prevention when I realise that there are mosquitoes around
20	I take measures for mosquito bite prevention when I visit places that mosquitoes might be present
21	I take measures for mosquito bite prevention every day during the daylight hours
22	I take measures for mosquito bite prevention every day during the evening and at night
23	I wear covering clothes to prevent mosquito bites
24	I use repellents to prevent mosquito bites
25	I use mosquito coil / repellent liquid vaporizers/tablet to keep mosquitoes away
